# 1,2-Bis(3-hydroxy­benzyl­idene)diazane

**DOI:** 10.1107/S1600536809027652

**Published:** 2009-07-18

**Authors:** Ling Zhu, Xin-Hua Zhao

**Affiliations:** a School of Chemistry and Life Science, Maoming University, Maoming 525000, People’s Republic of China; b School of Public Health, Guangdong Pharmaceutical University, Guangzhou 510006, People’s Republic of China

## Abstract

The asymmetric unit of the title compound, C_14_H_12_N_2_O_2_, which was synthesized unexpectedly by refluxing an ethano­lic solution of isonicotinic hydrazide and 3-hydroxy­benzaldehyde, contains one half-mol­ecule with the center of the N—N bond lying on a crystallographic center of inversion. In the crystal structure, mol­ecules are linked by inter­molecular O—H⋯N hydrogen bonds into an infinite layer structure parallel to (110).

## Related literature

For general background to salicyclic aldehyde complexes, see: Zelewsky & von Knof (1999 ▶); Alam *et al.* (2003 ▶).
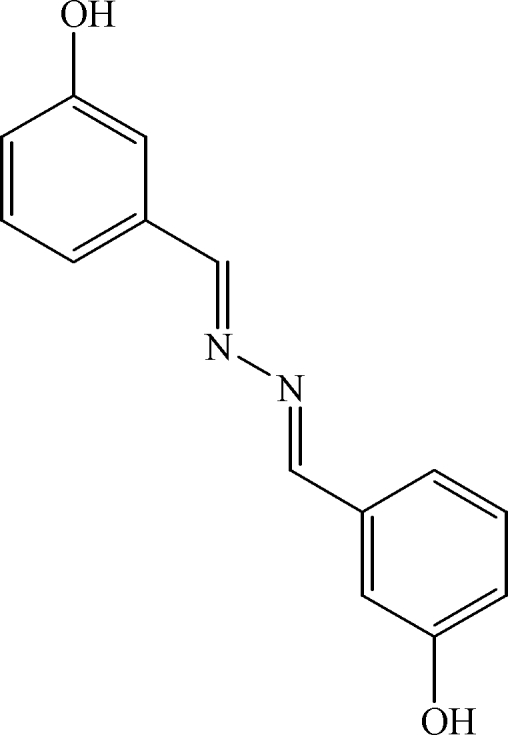


## Experimental

### 

#### Crystal data

C_14_H_12_N_2_O_2_
*M* *_r_* = 240.26
Monoclinic, 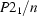

*a* = 4.883 (2) Å
*b* = 8.212 (3) Å
*c* = 14.575 (6) Å
β = 95.267 (6)°
*V* = 582.0 (4) Å^3^
*Z* = 2
Mo *K*α radiation
μ = 0.09 mm^−1^
*T* = 295 K
0.12 × 0.10 × 0.08 mm

#### Data collection

Bruker APEXII CCD area-detector diffractometer
Absorption correction: multi-scan (*SADABS*; Bruker, 2001 ▶) *T*_min_ = 0.989, *T*_max_ = 0.993
4234 measured reflections
1079 independent reflections
814 reflections with *I* > 2σ(*I*)
*R*_int_ = 0.039

#### Refinement

*R*[*F* ^2^ > 2σ(*F* ^2^)] = 0.064
*wR*(*F* ^2^) = 0.185
*S* = 1.00
1079 reflections
83 parameters
H-atom parameters not refined
Δρ_max_ = 0.62 e Å^−3^
Δρ_min_ = −0.23 e Å^−3^

### 

Data collection: *APEX2* (Bruker, 2004 ▶); cell refinement: *SAINT-Plus* (Bruker, 2001 ▶); data reduction: *SAINT-Plus*; program(s) used to solve structure: *SHELXS97* (Sheldrick, 2008 ▶); program(s) used to refine structure: *SHELXL97* (Sheldrick, 2008 ▶); molecular graphics: *SHELXTL* (Sheldrick, 2008 ▶); software used to prepare material for publication: *SHELXTL*.

## Supplementary Material

Crystal structure: contains datablocks I, global. DOI: 10.1107/S1600536809027652/im2128sup1.cif

Structure factors: contains datablocks I. DOI: 10.1107/S1600536809027652/im2128Isup2.hkl

Additional supplementary materials:  crystallographic information; 3D view; checkCIF report

## Figures and Tables

**Table 1:** Hydrogen-bond geometry (Å, °)

*D*—H⋯*A*	*D*—H	H⋯*A*	*D*⋯*A*	*D*—H⋯*A*
O1—H2⋯N1^i^	0.82	2.03	2.811 (3)	159

Symmetry code: (i) 
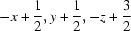
.

**Fig. 1.**
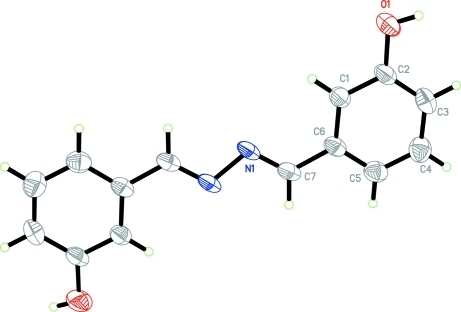
The molecular structure of (I), drawn with 50% probability displacement ellipsoids.

**Fig. 2.**
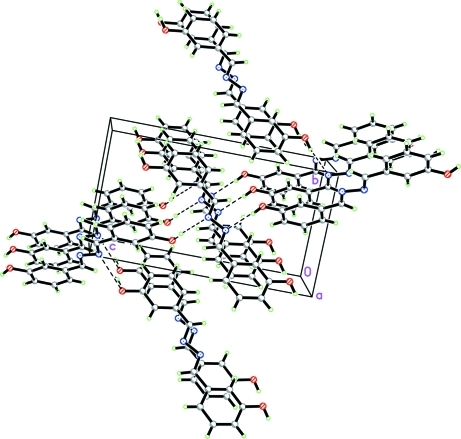
Three-dimensional network formed by hydrogen bonds (dashed lines).

## supplementary crystallographic information

### Comment

The synthesis of complexes consisting of ligands derived from salicylic aldehyde has attracted continuous research interest not only with regard to their appealing structural and topological novelty, but also due to their unusual optical, electronic, magnetic and catalytic properties as well as their potential medical application (Zelewsky *et al. *1999; Alam *et al.* 2003). In the present paper, we describe the synthesis and structural characterization of *N*,*N*'-di(3-hydroxybenzylidene)-hydrazine.

As shown in Fig. 1, the asymmetrical unit contains one half of the molecule. The center of the N-N bond represents a crystallographic center of inversion. One intermolecular hydrogen bond O(1)—H(2)···N(1) (2.811 (3) Å) is observed in the crystal structure leading to infinite layers of molecules (Fig. 2).

### Experimental

An ethanolic solution of isonicotinic hydrazide (10 mmol) and 3-hydroxybenzaldehyde (10 mmol) refluxed for five hours. After filtration a yellow powder was obtained. Suitable crystals for X-ray diffraction were obtained by recrystallization from dichloromethane. Anal. Calc. for C_14_H_12_N_2_O_2_: C 69.92, H 4.99, N 9.99%; Found: C 69.89, H 4.79, N 9.78.

### Refinement

All H atoms were placed in calculated positions with C—H = 0.93Å and refined as riding with *U*_iso_(H) = 1.2*U*_eq_(carrier). The H atom of the hydroxy group was located from difference density maps and was refined with a distance restraint of d(O–H) = 0.82 (1) Å.

### Crystal data

**Table:**

C_14_H_12_N_2_O_2_	*F*(000) = 252
*M**_r_* = 240.26	*D*_x_ = 1.371 Mg m^−^^3^
Monoclinic, *P*2_1_/*n*	Mo *K*α radiation, λ = 0.71073 Å
Hall symbol: -P 2yn	Cell parameters from 1079 reflections
*a* = 4.883 (2) Å	θ = 2.8–25.5°
*b* = 8.212 (3) Å	µ = 0.09 mm^−^^1^
*c* = 14.575 (6) Å	*T* = 295 K
β = 95.267 (6)°	Block, yellow
*V* = 582.0 (4) Å^3^	0.12 × 0.10 × 0.08 mm
*Z* = 2	

### Data collection

**Table:**

Bruker APEXII CCD area-detector diffractometer	1079 independent reflections
Radiation source: fine-focus sealed tube	814 reflections with *I* > 2σ(*I*)
graphite	*R*_int_ = 0.039
φ and ω scans	θ_max_ = 25.5°, θ_min_ = 2.8°
Absorption correction: multi-scan (*SADABS*; Bruker, 2001)	*h* = −5→5
*T*_min_ = 0.989, *T*_max_ = 0.993	*k* = −9→9
4234 measured reflections	*l* = −17→17

### Refinement

**Table:**

Refinement on *F*^2^	Primary atom site location: structure-invariant direct methods
Least-squares matrix: full	Secondary atom site location: difference Fourier map
*R*[*F*^2^ > 2σ(*F*^2^)] = 0.064	Hydrogen site location: inferred from neighbouring sites
*wR*(*F*^2^) = 0.185	H-atom parameters not refined
*S* = 1.00	*w* = 1/[σ^2^(*F*_o_^2^) + (0.103*P*)^2^ + 0.3322*P*] where *P* = (*F*_o_^2^ + 2*F*_c_^2^)/3
1079 reflections	(Δ/σ)_max_ < 0.001
83 parameters	Δρ_max_ = 0.62 e Å^−^^3^
0 restraints	Δρ_min_ = −0.23 e Å^−^^3^

### Special details

**Table:**

Geometry. All e.s.d.'s (except the e.s.d. in the dihedral angle between two l.s. planes) are estimated using the full covariance matrix. The cell e.s.d.'s are taken into account individually in the estimation of e.s.d.'s in distances, angles and torsion angles; correlations between e.s.d.'s in cell parameters are only used when they are defined by crystal symmetry. An approximate (isotropic) treatment of cell e.s.d.'s is used for estimating e.s.d.'s involving l.s. planes.
Refinement. Refinement of *F*^2^ against ALL reflections. The weighted *R*-factor *wR* and goodness of fit *S* are based on *F*^2^, conventional *R*-factors *R* are based on *F*, with *F* set to zero for negative *F*^2^. The threshold expression of *F*^2^ > σ(*F*^2^) is used only for calculating *R*-factors(gt) *etc*. and is not relevant to the choice of reflections for refinement. *R*-factors based on *F*^2^ are statistically about twice as large as those based on *F*, and *R*- factors based on ALL data will be even larger.

### Fractional atomic coordinates and isotropic or equivalent isotropic displacement parameters (Å^2^)

**Table:**

	*x*	*y*	*z*	*U*_iso_*/*U*_eq_	
C1	0.3137 (6)	0.2100 (3)	0.82062 (17)	0.0364 (7)	
H3	0.1725	0.1396	0.7996	0.044*	
C2	0.4468 (6)	0.2997 (3)	0.75848 (18)	0.0380 (7)	
C3	0.6607 (6)	0.4021 (4)	0.7888 (2)	0.0434 (8)	
H6	0.7546	0.4600	0.7467	0.052*	
C4	0.7340 (6)	0.4175 (4)	0.8822 (2)	0.0469 (8)	
H9	0.8749	0.4882	0.9031	0.056*	
C5	0.6000 (6)	0.3292 (4)	0.94433 (19)	0.0414 (8)	
H7	0.6516	0.3401	1.0070	0.050*	
C6	0.3891 (6)	0.2240 (3)	0.91470 (17)	0.0342 (7)	
C7	0.2426 (6)	0.1379 (3)	0.98224 (18)	0.0367 (7)	
H4	0.2836	0.1634	1.0441	0.044*	
N1	0.0615 (5)	0.0295 (3)	0.96142 (14)	0.0358 (6)	
O1	0.3545 (6)	0.2849 (3)	0.66792 (14)	0.0637 (8)	
H2	0.4174	0.3590	0.6386	0.096*	

### Atomic displacement parameters (Å^2^)

**Table:**

	*U*^11^	*U*^22^	*U*^33^	*U*^12^	*U*^13^	*U*^23^
C1	0.0469 (16)	0.0311 (14)	0.0322 (15)	0.0014 (12)	0.0096 (12)	0.0011 (11)
C2	0.0528 (17)	0.0348 (15)	0.0280 (14)	0.0046 (13)	0.0126 (12)	0.0034 (11)
C3	0.0420 (16)	0.0442 (17)	0.0463 (18)	0.0016 (13)	0.0157 (13)	0.0103 (14)
C4	0.0448 (17)	0.0431 (17)	0.0520 (18)	−0.0014 (14)	−0.0003 (14)	0.0064 (14)
C5	0.0475 (17)	0.0418 (17)	0.0343 (15)	0.0059 (14)	0.0004 (13)	0.0027 (12)
C6	0.0413 (15)	0.0338 (15)	0.0286 (13)	0.0092 (12)	0.0095 (11)	0.0029 (11)
C7	0.0501 (17)	0.0369 (16)	0.0241 (13)	0.0076 (13)	0.0085 (12)	0.0016 (11)
N1	0.0514 (14)	0.0354 (12)	0.0228 (11)	0.0081 (11)	0.0150 (9)	0.0040 (9)
O1	0.105 (2)	0.0549 (15)	0.0329 (12)	−0.0206 (14)	0.0170 (12)	0.0046 (10)

### Geometric parameters (Å, °)

**Table:**

C1—C2	1.376 (4)	C4—H9	0.9300
C1—C6	1.392 (4)	C5—C6	1.382 (4)
C1—H3	0.9300	C5—H7	0.9300
C2—O1	1.360 (3)	C6—C7	1.453 (4)
C2—C3	1.382 (4)	C7—N1	1.272 (4)
C3—C4	1.382 (4)	C7—H4	0.9300
C3—H6	0.9300	N1—N1^i^	1.409 (4)
C4—C5	1.372 (4)	O1—H2	0.8200
			
C2—C1—C6	120.4 (3)	C4—C5—C6	120.7 (3)
C2—C1—H3	119.8	C4—C5—H7	119.6
C6—C1—H3	119.8	C6—C5—H7	119.6
O1—C2—C1	117.2 (3)	C5—C6—C1	118.8 (3)
O1—C2—C3	122.5 (3)	C5—C6—C7	119.4 (2)
C1—C2—C3	120.3 (3)	C1—C6—C7	121.7 (3)
C2—C3—C4	119.4 (3)	N1—C7—C6	123.6 (2)
C2—C3—H6	120.3	N1—C7—H4	118.2
C4—C3—H6	120.3	C6—C7—H4	118.2
C5—C4—C3	120.4 (3)	C7—N1—N1^i^	112.8 (3)
C5—C4—H9	119.8	C2—O1—H2	109.5
C3—C4—H9	119.8		

Symmetry codes: (i) −*x*, −*y*, −*z*+2.

### Hydrogen-bond geometry (Å, °)

**Table:**

*D*—H···*A*	*D*—H	H···*A*	*D*···*A*	*D*—H···*A*
O1—H2···N1^ii^	0.82	2.03	2.811 (3)	159

Symmetry codes: (ii) −*x*+1/2, *y*+1/2, −*z*+3/2.
